# Successful Off-Label Use of Recombinant Factor VIIa and Coil Embolization in an Adolescent with Massive Hemoptysis Due to Invasive Pulmonary Aspergillosis

**DOI:** 10.4274/tjh.2014.0347

**Published:** 2015-02-15

**Authors:** Dilek Gürlek Gökçebay, Ali Fettah, İsmail Kırbaş, Bahattin Tunç, Namık Yaşar Özbek

**Affiliations:** 1 Ankara Children’s Hematology and Oncology Hospital, Clinic of Pediatric Hematology, Ankara, Turkey; 2 Turgut Özal University Faculty of Medicine, Department of Interventional Radiology, Ankara, Turkey

**Keywords:** Invasive pulmonary aspergillosis, Recombinant factor VIIa, Coil embolization, children, Acute leukemia

## Abstract

Invasive fungal infections have turned out to be a significant cause of morbidity and mortality in pediatric patients with malignant disorders. Massive hemoptysis, a rare complication of invasive pulmonary aspergillosis, may threaten the lives of patients, usually during the resolution of neutropenia. In this report, we describe a patient with massive hemoptysis due to invasive pulmonary aspergillosis whose bleeding was controlled successfully with off-label use of recombinant factor VIIa and subsequent coil embolization of the right pulmonary artery.

## Abstract (tr)

İnvaziv fungal enfeksiyonlar pediatrik malin hastalıklarda morbidite ve mortalitenin önemli bir nedenidir. Masif hemoptizi de sıklıkla nötropenin düzelme aşamasında görülen, invaziv pulmoner aspergillozisin hayatı tehdit edici nadir bir komplikasyonudur. Bu yazıda invaziv pulmoner aspergillozise bağlı masif hemoptizi gelişen bir hastada endikasyon dışı recombinant FVIIa kullanımı ardından sağ pulmoner artere coil embolizasyon uygulanarak kanama kontrolu sağlanan bir olgu sunulmuştur.

## INTRODUCTION

The incidence of invasive fungal infections is increasing worldwide in patients with acute leukemia due to the use of more aggressive chemotherapy regimens that result in severe neutropenia [1,2]. Despite the availability of new antifungal agents, the global death rate remains quite high because of the difficulty in establishing diagnoses [3]. Invasive pulmonary aspergillosis (IPA) is a severe mycosis characterized by acute invasion of the pulmonary vessels, leading to local parenchymal destruction, hemorrhage, thrombosis, and widespread hematogenous dissemination [4]. Massive hemoptysis is a life-threatening complication of IPA as we reported previously [5]. We herein report another adolescent patient with massive hemoptysis treated successfully with off-label use of recombinant factor VIIa (rFVIIa) and subsequent coil embolization of the right pulmonary artery. To the best of our knowledge, this is the first report concerning transarterial coil embolization for control of massive life-threatening hemoptysis in children with IPA. Informed consent was obtained.

## CASE PRESENTATION

A 16-year-old previously healthy girl was admitted to our hospital with ongoing menorrhagia for the past 2 weeks and headache and vomiting for the past 2 days. Her physical examination revealed normal findings except for signs of meningeal irritation. Complete blood count revealed hemoglobin of 8.1 g/dL, white blood cell count of 64.4x109/L, and platelet count of 70x109/L. Her peripheral blood and bone marrow aspiration smears showed FAB L2-type lymphoblasts. Blastic infiltration was evident in her cerebrospinal fluid examination. Bone marrow cytogenetic analysis revealed hypodiploidy in 2 of the 17 metaphases. She was diagnosed with acute lymphoblastic leukemia (ALL) with central nervous system involvement, and ALL-IC BFM 2009 induction therapy was initiated.

**Figure 1:**
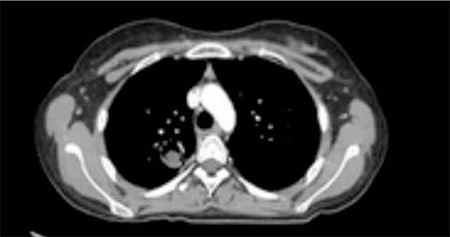
Thorax computed tomography with halo sign.

On follow-up, she had not achieved bone marrow remission at days 15, 33, or 52. She was diagnosed with resistant ALL and therefore received the IDA-FLAG regimen according to our institution’s decision (fludarabine at 30 mg/m2/day for 4 days; cytarabine at 2 g/m2/day for 4 days; idarubicin at 12 mg/m2/day on days 2, 3, and 4; and granulocyte-colony stimulating factor at 5 µg/kg/day). After the IDA-FLAG regimen, she developed prolonged neutropenic fever. Meanwhile, due to infiltration of the lungs on chest radiography, she was treated with broad-spectrum antibiotics. Due to persistent fever, computed tomography (CT) of the thorax was performed, revealing 2 nodules in the right lung surrounded by a halo of ground-glass attenuation. At this point, serum galactomannan antigen was also positive, and so, considering the existence of IPA, voriconazole was added to the treatment. After resolution of the neutropenic period, her bone marrow was found to be in remission and repeated thorax CT showed persistence of IPA with 2 cavities in the right lung (Figure 1). Thorax CT angiography showed no evidence of vessel invasion at this stage. Therefore, she received the FLAG protocol under voriconazole treatment.

**Figure 2:**
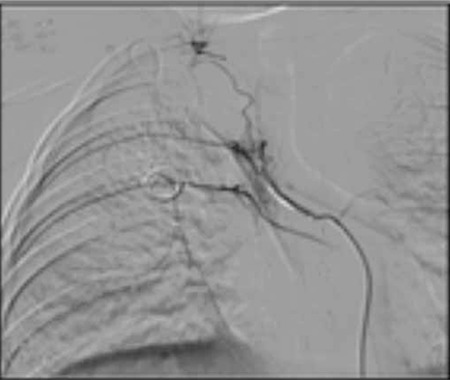
Coil embolization of the right bronchial artery.

One week after the FLAG treatment, at day 37 of the voriconazole treatment, she developed massive hemoptysis with coughing during a thrombocytopenic period (platelet count: 9x109/L). Otolaryngological examination revealed bleeding from the subepiglottic area. Despite receiving adequate platelet and cryoprecipitate support, about 300 mL of fresh bleeding was observed within 90 min. During that time, her hemoglobin level dropped from 9.5 g/dL to 6.5 g/dL. Hemostasis was achieved with rFVIIa given 3 times at doses of 90 µg/kg at 2-h intervals. In the meantime, she received consultation from the interventional radiology department; the right bronchial artery was selectively embolized with a coil immediately after the last factor VIIa infusion (Figure 2). There was no recurrence during the 3 months of follow-up.

## DISCUSSION AND REVIEW OF THE LITERATURE

Hemoptysis may be caused by chronic inflammatory lung diseases including bronchiectasis, tuberculosis, and aspergillosis [6]. Massive hemoptysis has been described as the expectoration of an amount of blood ranging from 100 to >1000 mL over 24 h; in 90% of cases of massive hemoptysis, the bleeding originates from the bronchial arterial circulation. It is a medical emergency that requires prompt evaluation and management. Conservative management of massive hemoptysis carries a mortality rate of as high as 50%-85% [7,8,9].

Hemoptysis due to fungal invasion of the pulmonary vessels is reported at rates of up to 58% during the neutropenic period in patients with IPA [10]. However, massive hemoptysis usually occurs during the phase of bone marrow recovery, as in one of our previously reported patients [5]. The pathogenesis of this phenomenon is not fully understood. Chemotherapy-induced severe neutropenia results in an immunodeficient state facilitating infection with filamentous mycosis. Following recovery of the bone marrow, the neutrophils are chemoattracted to the lung regions infected with fungi that enhance the local inflammatory response and release of proteolytic enzymes. These may play roles in the invasion of blood vessels by filamentous mycosis [4,10,11].

The initial approach to treating life-threatening massive hemoptysis is maintenance of the airway and oxygenation. After stabilizing the hemodynamic status, topical application of antifibrinolytic agents, adrenaline, or thrombin-fibrinogen solutions into the bleeding bronchi via bronchoscopy can be performed; however, the efficacy of those treatments is uncertain [12]. rFVIIa has been widely used as an off-label drug for patients with critical bleeding. It acts with tissue factor and leads to thrombin generation via the extrinsic clotting pathway. It also activates factor X on the surface of activated platelets, which is called “thrombin burst” [13]. The main concern with the administration of rFVIIa is the potential for inappropriate thrombosis. In an earlier study, thromboembolic adverse events were reported at 5.3% in patients who received rFVIIa for refractory hemorrhage. No difference was found between placebo-treated and rFVIIa-treated patients for thromboembolic events [14]. In a case report concerning the successful use of rFVIIa in the treatment of an adult patient with massive life-threatening hemoptysis due to chronic necrotizing Aspergillus infection, 2 administrations of rFVIIa at doses of 30 µg/kg were used and the hemoptysis was successfully resolved without recurrence [15]. Similarly, another report revealed the utility of rFVIIa in the critical refractory bleeding of 2 adult patients [16]. We could not find a pediatric case in the English literature similar to that of our present patient who responded to treatment with factor VIIa.

Various embolic materials, such as polyvinyl alcohol particles, coils, and microspheres, have been used for selective bronchial and nonbronchial systemic arterial embolization in patients with hemoptysis [8]. The use of liquid agents, such as alcohol, is not recommended because of very fast delivery, possible tracheal or bronchial necrosis related to extremely distal occlusion of the bronchial arteries, and occlusion of collateral vessels [17,18]. On the other hand, using coils prevents repeat embolization if hemoptysis recurs. In addition, coils cause proximal occlusion, which promotes the development of new collateral circulation from the surrounding vessels [19]. Dohen-Bécue et al. reported 4 patients with massive hemoptysis due to IPA [20]. Two of them received embolization, but the procedure was only successful in one of them and the other patient died. In our case, 3 doses of rFVIIa (90 µg/kg) were enough to control the massive bleeding. However, due to continuing leakage, we could not exclude a recurrence; therefore, selective right bronchial arterial coil embolization was performed. No complications or recurrent hemoptysis occurred.

To the best of our knowledge, this is the first pediatric case in the English literature of massive hemoptysis due to IPA treated successfully with the aid of rFVIIa infusion and transarterial coil embolization. In conclusion, in emergency circumstances, we suggest off-label use of rFVIIa to control bleeding, followed by coil embolization to achieve durable hemostasis if necessary.
